# Identification and Expression Profiling of Odorant Binding Proteins and Chemosensory Proteins between Two Wingless Morphs and a Winged Morph of the Cotton Aphid *Aphis gossypii* Glover

**DOI:** 10.1371/journal.pone.0073524

**Published:** 2013-09-20

**Authors:** Shao-Hua Gu, Kong-Ming Wu, Yu-Yuan Guo, Linda M. Field, John A. Pickett, Yong-Jun Zhang, Jing-Jiang Zhou

**Affiliations:** 1 State Key Laboratory for Biology of Plant Diseases and Insect Pests, Institute of Plant Protection, Chinese Academy of Agricultural Sciences, Beijing, China; 2 Department of Biological Chemistry and Crop Protection, Rothamsted Research, Harpenden, United Kingdom; University College Dublin, Ireland

## Abstract

Insects interact with their environment and respond to the changes in host plant conditions using semiochemicals. Such ecological interactions are facilitated by the olfactory sensilla and the use of olfactory recognition proteins. The cotton aphid *Aphis gossypii* can change its phenotype in response to ecological conditions. They reproduce mainly as wingless asexual morphs but develop wings to find mates or new plant hosts under the influence of environmental factors such as temperature, plant nutrition and population density. Two groups of small soluble proteins, odorant binding proteins (OBPs) and chemosensory proteins (CSPs) are believed to be involved in the initial biochemical recognition steps in semiochemical perception. However, the exact molecular roles that these proteins play in insect olfaction remain to be discovered. In this study, we compared the transcriptomes of three asexual developmental stages (wingless spring and summer morphs and winged adults) and characterised 9 OBP and 9 CSP genes. The gene structure analysis showed that the number and length of introns in these genes are much higher and this appears to be unique feature of aphid OBP and CSP genes in general. Another unique feature in aphids is a higher abundance of CSP transcripts than OBP transcripts, suggesting an important role of CSPs in aphid physiology and ecology. We showed that some of the transcripts are overexpressed in the antennae in comparison to the bodies and highly expressed in the winged aphids compared to wingless morphs, suggesting a role in host location. We examined the differential expression of these olfactory genes in ten aphid species and compared the expression profile with the RNA-seq analyses of 25 pea aphid transcriptome libraries hosted on AphidBase.

## Introduction

Insects use sensitive olfactory systems to detect airborne chemicals from the environment and to find preferred hosts, mates and oviposition sites [1-4]. The sap-sucking aphids are destructive pests of many economically important crops throughout the world. Like other insects, aphids use chemical molecules such as species-specific pheromones and plant volatiles to interact with each other, host plants and to react to changes in their environment. Mature sexual females of many aphid species release a mixture of two iridoids (*4aS*, *7S*, *7aR*)-nepetalactone and (*1R*, *4aS*, *7S*, *7aR*)-nepetalactol which act as sex pheromones to attract conspecific males [5,6]. Another semiochemical which is widely used by most aphid species is the alarm pheromone (*E*)-β-farnesene which warns neighbouring aphids of attacks and overcrowding [7,8]. (E) -β-farnesene is also used as a foraging cue for many of the aphids’ natural enemies [6,8]. Many plants release (*E*)-β-farnesene as a component of their essential oils. To avoid responding to this compound when not released by aphids, there are specific olfactory neurons co-located with (*E*)-β-farnesene neurons, for other sesquiterpenes such as (1*R*,4*E*,9*S*)-caryophyllene in aphids [9,10] and also in the typical aphid predators *Coccinellaseptempunctata* [11]. The combinatory actions of these neurons in aphids allow them to discriminate (*E*)-β-farnesene released by plants and aphids. Plants release aphid-induced defence volatiles to attract aphid predators and parasitoids [12,13]. Aphids use plant volatiles to locate suitable hosts and to avoid unfavourable plants by detecting chemical signals emitted by plants in response to aphid feeding and nutrient condition. Aphids are specifically sensitive to the homoterpenes such as (*E*)-4,8-dimethyl-1,3,7-nonatriene and (*E*, E)- 4,8,12-trimethyltrideca-1,3,7,11-tetraene which are produced by plants attacked by aphids and which reduce colonisation or attraction of predators or parasitoids in cotton aphids [14] and other aphids [15]. Thus, studying how aphid’s respond to pheromones and plant volatiles at the molecular level offers promising ways to explain the ecological context of aphid-aphid and aphid-plant interactions. In turn, this will facilitate the design and implementation of novel sustainable aphid management strategies for pest control and benefit environmental and ecological systems.

Two families of small soluble proteins, odorant binding proteins (OBPs) and chemosensory proteins (CSPs) are concentrated (as high as 10 mM) in the sensillum lymph of the antennae of insects and are thought to be involved in chemosensory perception [16-19]. Both OBPs and CSPs are considered as carrier proteins, taking part in the initial biochemical recognition steps of odorant perception by capturing and transporting hydrophobic odorant molecules across the aqueous lumen of the antennae to membrane-bound olfactory receptors (ORs) [18,19,20-22]. To date studies on the involvement of OBPs and CSPs in aphid olfaction are limited and sometimes contradictory. Ligand binding assays have suggested that OBP3 of the pea aphid *Acyrthosiphon pisum* and OBP7 of the wheat aphid *Sitobion* *avenae* have high binding affinity with (*E*)-β-farnesene [23,24]. However, for the vetch aphid *Megouraviciae* where two CSPs MvicOS-D1 and MvicOS-D2 were identified no binding could be shown for any of twenty-eight compounds known to elicit an electrophysiological response in electroantennograms or in single olfactory neurone preparations [25]. Recent publication of the genome sequence of *A* *. pisum* has facilitated the annotation of putative OBPs and CSPs in *A* *. pisum* and in turn this has allowed the identification of OBPs and CSPs in other aphid species [26,27].

**Figure 1:**
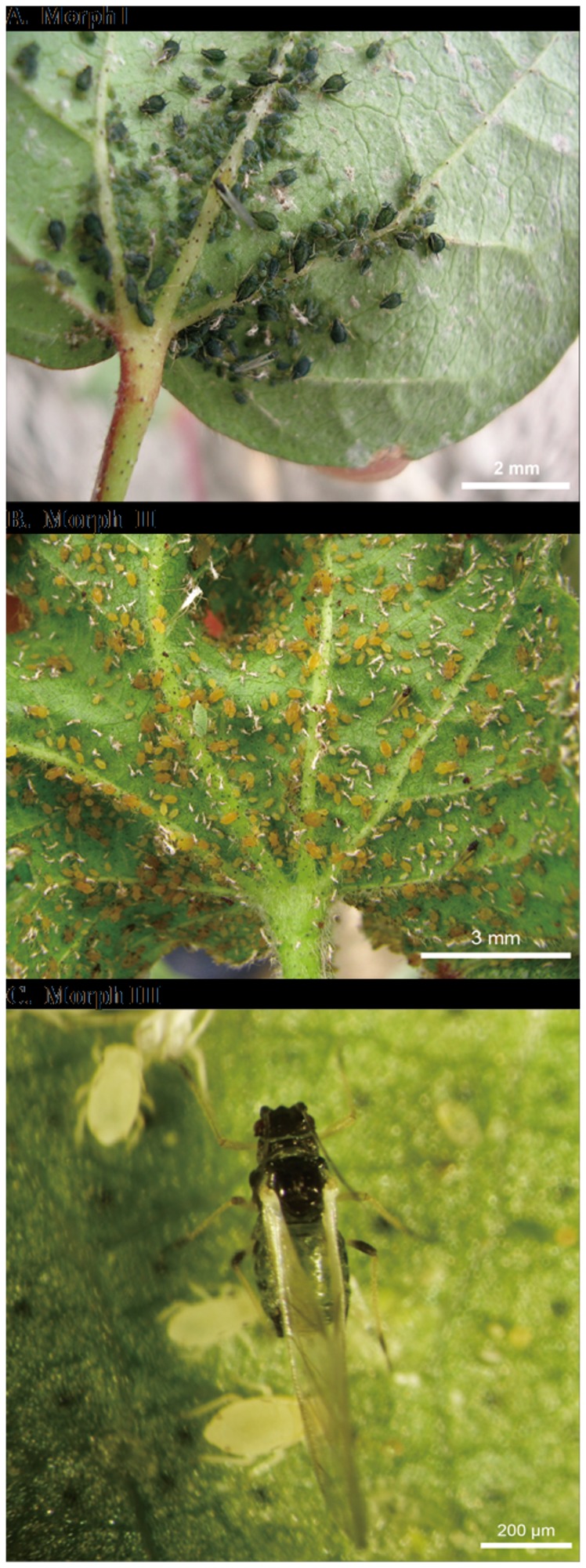
Three morphs of *A. gossypii* used in this study. **A**. Morph I: wingless asexual aphids which occur on cotton plants at 16°C ~ 24°C in spring. **B**. Morph II: wingless asexual aphids which occur on cotton plants at 24°C ~ 27°C in summer. **C**. Morph III: winged adults produced from Morph I or Morph II under conditions of poor host plant nutrition, crowded populations or unfavorable environmental conditions.

The cotton aphid *Aphis gossypii* is a polyphagous pest of cotton, melon and other plant species, transmitting more than 80 virus diseases, including banana mosaic, papaya mosaic, papaya ring spot, citrus tristeza and passion fruit woody virus. On cotton plants, *A* *. gossypii* can exist as three ecologically important developmental stages. Under the right climatic conditions, there are two wingless (apterous) forms (Morph I, Morph II) and a winged (alate) form (Morph III) (Figure 1). The aphids in each morph adapt to specific environmental conditions and exhibit phenotypic differences due to environmental heterogeneity. Morph I, with a larger body size and darker color (usually dark green or black) is found on seedlings and young cotton plants, where they reproduce parthenogenetically and cause direct feeding damage. Morph II are again asexual but are smaller and light green in colour and are found on older plants during the summer where they resist high temperatures and have a high fecundity resulting in high levels of feeding damage. When the population becomes too crowded and the cotton crops are less nutritious or when there are unfavorable environmental conditions a third type Morph III, a dark bluish-green winged adult arises. This leaves the cotton plant, and either returns to its primary tree host, where sexual forms arise and mate to produce fertilized eggs for overwintering or disperses to other plants where nutritional or ecological conditions are more suitable to produce young that grow into wingless adults. These behaviours are mediated by chemical cures such as the sex pheromones, the alarm pheromone and plant volatiles [6].

In the present study, we produced the transcriptomes of three morphs of the cotton aphid *A* *. gossypii* , identified OBP and CSP genes and for the first time experimentally demonstrated the genomic structure of these aphid genes, with uniquely long introns. We then determined their expression levels in different tissues and in three ecologically different life forms by quantitative real-time PCR. This has provided rich resources for further functional characterization of the *A. gosypii* OBPs/CSPs. We consider the potential role of OBPs/CSPs in determining olfactory responses in three different morphs and the relevance to the ecological systems in which they exist. The evolutionary relationships of aphid OBP and CSP proteins are also discussed.

## Materials and Methods

### Aphid collection and rearing

*A* *. gossypii* were collected from a cotton field at Langfang Experimental Station of the Chinese Academy of Agricultural Sciences, Hebei Province, China in 2011 and a single female was used to establish the experimental colony which produces a population composed of genetically identical individuals. The colony was reared on cotton seedlings in chambers, at 18-24°C, 65-75% RH, under a 16h: 8h light:dark photo regime with aphids being transferred to new cotton seedlings each week.

Aphids for RNA and genomic DNA extraction and for transcriptome sequencing were obtained for each of the three Morphs by rearing under different conditions. Morph I were raised from newly emerged nymphs at 16-24°C. Morph II were obtained from Morph I by moving to 24-27°C and Morph III were reared from Morph II at 24-27°C under crowded conditions.

About 40 mg of aphids of each Morph were collected into a 1.5 ml centrifuge tube and kept in liquid nitrogen until use. For the tissue studies about 2000 Morph III aphids were dissected on ice under magnification and the antennae and the decapitated body parts were collected separately in tubes and immediately frozen in liquid nitrogen and stored at -80°C.

### Transcriptome sequencing

Total RNAs were extracted using TRIzol regent (Invitrogen, Carlsbad, CA, USA) from each of three aphid Morphs according to the manufacturer’s protocol. About 500 ng mRNA was purified from 50 μg total RNA using polyATtract mRNA isolation system III (Promega, Madison, WI, USA). The cDNA library construction and the 454 GS FLX sequencing were conducted at Autolab Biotechnology Company (Beijing, China). After sequencing, the raw 454 reads were processed to remove low quality and adaptor sequences and assembled into unigenes using Mimicking Intelligent Read Assembly MIRA3 [28] and Contig Assembly Program CAP3 [29].

### Identification of transcripts encoding putative OBPs and CSPs

Two methods were used to identify unigenes encoding putative *OBPs* and *CSPs* in each of three aphid Morphs(1). The “OBP MotifSearch program” of C1-X_15-39_-C2-X_3_-C3-X_21-44_-C4-X_7-12_-C5-X_8_-C6 [30] and the “CSP MotifSearch program” of C_1_-X_6-8_-C_2_-X_16-21_-C_3_-X_2_-C_4_ [31] were performed to identify putative OBP and CSP genes, respectively(2). tBLASTn was performed, using known OBP and CSP sequences from other aphid species as the “query”. All candidate OBP and CSP genes were manually checked using the BLASTx program available at the National Center for Biotechnology Information (NCBI) and sequencing.

### Transcript abundance analysis of the transcriptome dataset

The abundances of the unigenes in the transcriptomes were calculated by the RPKM (Reads Per Kilobase per Million mapped reads) method, using the formula: RPKM (A) = (1,000,000×C×1,000) / (N×L), where RPKM (A) is the expression abundance of gene A; C is the number of reads that are uniquely mapped to gene A; N is the total number of reads that are uniquely mapped to all genes and L is the number of bases on gene A. The RPKM method is able to eliminate the influence of different gene lengths and sequencing discrepancies on the calculation of transcript abundance.

### Verification of OBP and CSP sequences by cloning and sequencing

Open reading frames (ORFs) of each identified OBP and CSP sequence were found by ORF Finder graphical analysis at NCBI (http://www.ncbi.nlm.nih.gov/gorf/gorf.html). Then gene-specific primers were designed and used to clone the ORF sequence of each OBP and CSP gene (Table S1). Template cDNA was synthesized using the SuperScript^TM^ III Reverse Transcriptase system (Invitrogen, Carlsbad, CA). PCR reactions were carried out with 200 ng antennal cDNAs with 0.5 units of *Ex Taq* DNA Polymerase (TaKaRa, Dalian, China) and cycling conditions were: initial denaturation at 95°C for 3 min; then 36 cycles of 94°C for 45 sec, 56°C for 1 min, 72°C for 1 min, and final extension at 72°C for 10 min. The PCR products were gel-purified and subcloned into the pMD 19-T simple vector (TaKaRa, Dalian, China) and the inserts were sequenced using standard M13 primers at Beijing Genomic Institute (Beijing, China).

### Analysis of OBP and CSP genomic structures

Genomic DNA from about 40 mg whole-bodies of Morph III aphids was extracted using E.Z.N.A. Insect DNA Kit (Omega Bio-Tek, Norcross, USA) following the manufacturer’s instructions. Gene-specific primer combinations and *LA Taq* DNA Polymerase (TaKaRa, Dalian, China) were used to amplify the genomic DNA sequence of each OBP and CSP gene. LA PCR (Long and Accurate PCR) cycling program was conducted as follows: initial denaturation at 95°C for 2 min; then 35 cycles of 98°C for 10 sec, 68°C for 2~10 min (depending on the target gene length); and a final extension step at 72°C for 10 min. The PCR products were gel purified using QIAquick Gel Extraction Kit (Qiagen, Hilden, Germany) and ligated into the pGEM-T easy vector (Promega, Madison, WI) and sequenced using SP6 and T7 primers in both directions. The mRNA-to-genomic DNA alignment of each OBP and CSP gene was analysed using the Spidey program (http://www.ncbi.nlm.nih.gov/IEB/Research/Ostell/Spidey/spideyweb.cgi).

### Analysis of expression levels of OBPs and CSPs in different Morphs and tissues

Total RNA from each of the three aphid Morphs and different tissues (antennae and decapitated bodies) of Morph III were extracted using TRIzol reagent (Invitrogen, Carlsbad, CA, USA). Before transcription, total RNA was treated with RQ1 RNase-Free DNase (Promega, Madison, USA) to remove residual genomic DNA. Single-stranded cDNAs were synthesized using the GoScript Reverse Transcription system (Promega, Madison, USA).

Quantitative real-time PCR (qRT-PCR) was carried out to assess the expression level of each OBP and CSP transcript in the three Morphs, and in different tissues (antenna and body). Specific primer pairs for qRT-PCR were designed with Primer 3 (http://frodo.wi.mit.edu/) (Table S2). qRT-PCR analysis was conducted using ABI 7500 Real-Time PCR System (Applied Biosystems, Carlsbad, CA). Two reference genes, *β-actin* and *18S ribosomal RNA* were used for normalizing the target gene expression and correcting for sample-to-sample variation. qRT-PCR reactions were done in 25 μl reactions containing 12.5 μl of SuperReal PreMix Plus (TianGen, Beijing, China), 0.75 μl of each primer (10 μM), 0.5 μl Rox Reference Dye, 1 μl sample cDNA (150 ng/μl), 9.5 μl sterilized H_2_O. The qRT-PCR cycling parameters were: 95°C for 15 min, followed by 40 cycles of 95°C for 10 sec and 60°C for 32 sec. Then, the PCR products were heated to 95°C for 15 sec, cooled to 60°C for 1 min and heated to 95°C for 30 sec and cooled to 60°C for 15 sec to measure the dissociation curves. Negative controls without either template or transcriptase were included in each experiment. To check reproducibility, each qRT-PCR reaction for each sample was carried out in three technical replicates and two biological replicates for each transcript. Relative quantification was performed using the comparative 2^-ΔΔCT^ method [32]. The comparative analyses were conducted with *t*-tests between each transcript expression in various tissues and with one-way nested analysis of variance (ANOVA) between developmental stages, followed by a Tukey’s honestly significance difference (HSD) test using SPSS Statistics 18.0 (SPSS Inc., Chicago, IL, USA). When applicable, values were presented as mean ± SE (two biological replicates combined with three technical replicates per biological replicate) for each transcript in one condition.

### Sequence analysis and phylogenetic tree construction

The putative N-terminal signal peptides and most likely cleavage site were predicted by the SignalP 4.0 Server [33] (http://www.cbs.dtu.dk/services/SignalP/). Sequence alignments were performed using ClustalX 2.1 [34] with default gap penalty parameters of gap opening 10 and extension 0.2 and were edited with GeneDoc 2.7.0 software. Identity values were calculated using Vector NTI Advance 11 software (Invitrogen Corporation, Carlsbad, CA). Phylogenetic trees were constructed by the neighbor joining method as implemented in PHYLIP package (Version 3.69 http://evolution.genetics.washington.edu/phylip.html) or MEGA5. Bootstrap support of tree branches was assessed by re-sampling amino acid positions 1000 times.

## Results and Discussion

### Identification of OBP and CSP genes in *A* *. gossypii*

We carried out next generation sequencing of cDNA libraries from three Morphs of *A* *. gossypii* : wingless (apterous) Morph I, wingless (apterous) Morph II and winged adults (alate) Morph III (Figure 1). A total of 54,547 unigenes were assembled from 1,113,269 clean reads and 9 putative OBP and 9 putative CSP transcripts identified (Table 1) using Motifsearch [35] and BLAST. We name the OBP genes as *AgosOBP2-10* and the CSP genes as *AgosCSP1-2* and *4-10* following the nomenclature established for *A* *. pisum* [27]. All of these OBP and CSP transcripts were confirmed by molecular cloning and sequencing. Among the 9 AgosOBPs, 8 have the characteristic insect OBP sequence motif “C1-X_15-39_-C2-X_3_-C3-X_21-44_-C4-X_7-12_-C5-X_8_-C6” [30] and the ninth OBP AgosOBP4 has 47 amino acids between the first and second conserved cysteines (Figure S1). All of the 9 CSP sequences have the insect CSP motif “C_1_-X_6-8_-C_2_-X_16-21_-C_3_-X_2_-C_4_” [31] (Figure S2). The 9 OBPs share 9%-24% amino acid identities with each other (Table S3), whilst the 9 CSPs share 16%-38% amino acid identities with each other (Table S4). The full-length sequences of the 9 AgosOBPs and 9 AgosCSPs have been deposited in GenBank under the accession numbers KC151555 to KC161572.

**Table 1:** OBPs and CSPs in *A. gossypii.*

Gene name	Accession Number	Number of Reads	Length (AA)	Signal peptide	Number of intron	Gene annotation	Homology search with known proteins				
							Species	Protein ID	Score (bits)	*E*-value	% Identity
AgosOBP2	KC161555	364	243	1-19 aa	4	odorant-binding protein 2	*Aphis craccivora*	CAR85658	391	2E-135	99%
AgosOBP3	KC161556	69	141	1-23 aa	5	odorant-binding protein 3	*Acyrthosiphon pisum*	NP_001153529	258	6E-86	86%
AgosOBP4	KC161557	35	198	1-22 aa	6	odorant-binding protein 4	*Acyrthosiphon pisum*	NP_001153530	340	1E-116	90%
AgosOBP5	KC161558	82	224	1-27 aa	8	odorant-binding protein 5	*Acyrthosiphon pisum*	NP_001153531	430	3E-151	91%
AgosOBP6	KC161559	196	215	1-19 aa	7	odorant-binding protein 6	*Acyrthosiphon pisum*	NP_001153532	272	5E-90	78%
AgosOBP7	KC161560	15	148	1-23 aa	6	odorant-binding protein 7	*Acyrthosiphon pisum*	NP_001153533	236	9E-77	81%
AgosOBP8	KC161561	26	161	1-18 aa	6	odorant-binding protein 8	*Acyrthosiphon pisum*	NP_001153534	305	1E-103	94%
AgosOBP9	KC161562	5	166	1-24 aa	6	odorant-binding protein 9	*Acyrthosiphon pisum*	NP_001153535	275	6E-92	84%
AgosOBP10	KC161563	6	147	1-24 aa	6	odorant-binding protein 10	*Acyrthosiphon pisum*	NP_001153525	220	8E-71	72%
AgosCSP1	KC161564	102	170	Not detected	1	chemosensory protein-like	*Acyrthosiphon pisum*	NP_001119650	278	4E-92	64%
AgosCSP2	KC161565	83	134	1-20 aa	1	chemosensory protein-like	*Acyrthosiphon pisum*	NP_001119651	227	5E-74	87%
AgosCSP4	KC161566	715	145	1-22 aa	1	chemosensory protein-like	*Acyrthosiphon pisum*	NP_001119652	256	4E-85	94%
AgosCSP5	KC161567	1121	139	1-19 aa	1	chemosensory protein CSP5	*Myzus* *persicae*	ACJ64049	241	3E-79	95%
AgosCSP6	KC161568	57	131	1-21 aa	1	chemosensory protein CSP1	*Myzus* *persicae*	ACJ64047	243	2E-80	87%
AgosCSP7	KC161569	86	152	1-24 aa	2	chemosensory protein 1-like	*Acyrthosiphon pisum*	NP_001156200	289	1E-97	90%
AgosCSP8	KC161570	193	162	1-37 aa	1	chemosensory protein CSP4	*Myzus* *persicae*	ACJ64048	254	8E-84	75%
AgosCSP9	KC161571	4	171	1-22 aa	1	chemosensory protein	*Artemiafranciscana*	ABY62738	71.2	4E-13	35%
AgosCSP10	KC161572	3	149	Not detected	2	chemosensory protein-like	*Acyrthosiphon pisum*	NP_001119649	105	3E-26	34%

Gene name, name of genes identified from *A* *. gossypii* . Length, number of amino acids including signal peptide region. Species, source species of homologous gene. Protein ID: specific number of homologs on public database. *E*-value, the statistical significance of reported matches. % Identities, Percentage of amino acid identities between *A* *. gossypii* and homologs.

### Sequence analysis and phylogenetic tree construction

**Figure 2:**
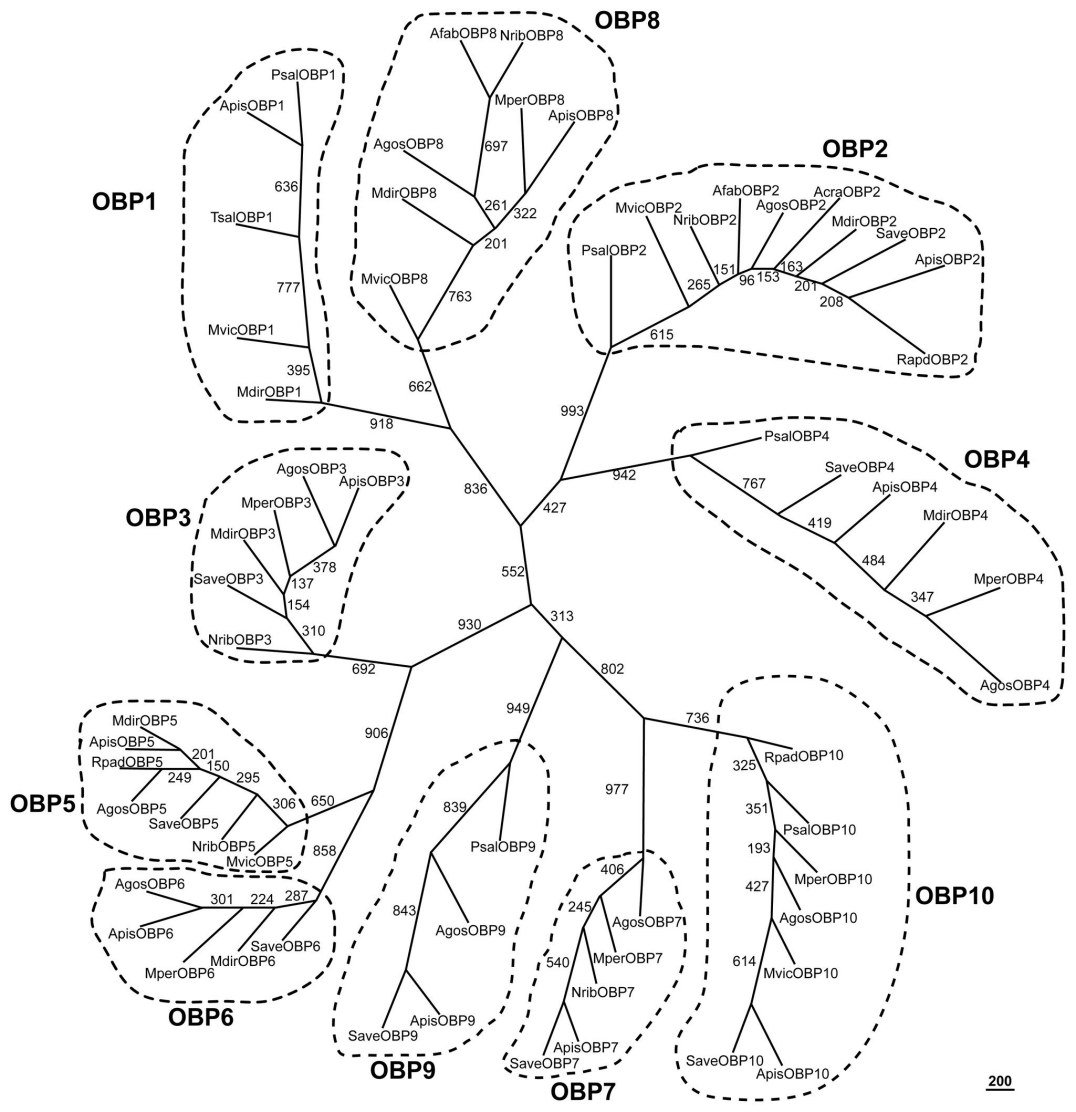
Phylogenetic tree of 62 OBPs from 12 aphid species. Numbers on branches show values of 1000 times replication bootstrap analysis and the bootstrap values are listed at each node. Accession numbers of the 62 aphid OBPs are as follow: Agos, *Aphis* *gossypii*. The accession numbers of AgosOBP2-10 are listed in Table 1. Psal, *Pterocomma* *salicis* (PsalOBP1: CAR85660; PsalOBP2: CAR85661; PsalOBP4: CAR85662; PsalOBP9: CAR85663; PsalOBP10: CAX63261); Afab, *Aphis* *fabae* (AfabOBP2: CAR85656; AfabOBP8: CAR85657); Acra, *Aphis* *craccivora* (AcraOBP2; CAR85658); Tsal, *Tuberolachnus* *salignus* (TsalOBP1: CAR85659); Mvic, *Megoura* *viciae* (MvicOBP1: CAR85650; MvicOBP2: CAR85651; MvicOBP5: CAR85652; MvicOBP8: CAR85653; MvicOBP10: CAX63260); Mdir, *Metopolophium* *dirhodum* (MdirOBP1: CAR85638; MdirOBP2: CAR85639; MdirOBP3: CAX63256; MdirOBP4: CAR85640; MdirOBP5: CAR85641; MdirOBP6: CAR85642; MdirOBP8: CAR85643); Mper, *Myzus* *persicae* (MperOBP3: CAR85644; MperOBP4: CAR85645; MperOBP6: CAR85646; MperOBP7: CAR85647; MperOBP8: CAR85648; MperOBP10: CAR85649); Nrib, *Nasonovia* *ribis-nigri* (NribOBP2: CAR85654; NribOBP3: CAX63257; NribOBP5: CAX63258; NribOBP7: CAX63259; NribOBP8: CAR85655). Save, *Sitobion* *avenae* (SaveOBP2: CAX63247; SaveOBP3: CAX63248; SaveOBP4: CAX63249; SaveOBP5: CAX63250; SaveOBP6: CAX63251; SaveOBP7: ACW03675; SaveOBP9: GQ847860; SaveOBP10: CAX63252); Rpad, *Rhopalosiphum* *padi* (RpadOBP2: CAX63253; RpadOBP5: CAX63254; RpadOBP10: CAX63255). Apis, *Acyrthosiphon* *pisum* (ApisOBP1-AipsOBP10: CAR85628-CAR85637).

Phylogenetic analysis of aphid OBPs revealed that these proteins cluster in 10 groups, each containing several homologous OBPs from different aphid species (Figure 2) with average amino acid identity of 81.2% within each group and 20.0% overall identity of 62 aphid OBPs. Phylogenetic analysis of CSPs from *A* *. gossypii* and *A* *. pisum* revealed that each CSP gene clustered into one branch with very high amino acid identities (59%-95%) between the two aphid species (Figure S3), consistent with a previous report of high conservation between aphid species [27]. The high identities of the OBPs in each group from different aphid species and the similarity of each pair of CSPs from *A* *. gossypii* and *A* *. pisum* clearly indicates that these genes have evolved from a common ancestral gene and diverged before aphid speciation. This may well have contributed to host plant adaptation and the use of different ratios of the sex pheromone components by each aphid species. It is interesting that we failed to find *A* *. gossypii* homologues of OBP1 and CSP3 during the *A* *. gossypii* transcriptome analysis and by RT-PCR with gene-specific primers of *A* *. pisum* which were used successfully to identify OBP homologs from other aphid species [27]. It is possible that in some cases OBP and CSP primers fail to detect closely related sequences in other aphids due to their low expression levels or the genes might have been lost. We examined this by PCR amplification of cDNAs from 10 aphid species using *A. pisum* gene-specific primers for OBPs and CSPs without signal peptide sequences. These failed to produce PCR products in some species (Table 2) despite high amino acid identities of each OBP group between aphid species (Figure 2) with average amino acid identity of 81.2% within each group and 20.0% overall identity of 62 aphid OBPs. Overall *A* *. gossypii* has homologues of all of the *A. pisum* OBPs except for OBP1. On the other hand, the black willow aphid *Tuberolachnussalignus* only has one homologue of OBP1 (Table 2). Further studies are now required to investigate these differences between aphid species in the context of gene loss, gene evolution, expression regulation and aphid host adaptations in ecological systems.

**Table 2:** RT-PCR of OBPs and CSPs in *A. gossypii* using *A. pisum* gene-specific premiers.

**Species**	**CSP1**	**CSP2**	**CSP3**	**CSP4**	**CSP5**	**CSP6**	**CSP7**	**CSP8**	**CSP9**	**CSP10**
*Acyrthosiphon pisum*	•	•	•	•	•	•	•	•	•	•
*Aphis gossypii*	•	•	**x**	•	•	•	•	•	•	•
**Species**	**OBP1**	**OBP2**	**OBP3**	**OBP4**	**OBP5**	**OBP6**	**OBP7**	**OBP8**	**OBP9**	**OBP10**
*Acyrthosiphon pisum*	•	•	•	•	•	•	•	•	•	•
*Aphis gossypii*	**x**	•	•	•	•	•	•	•	•	•
*Megouraviciae*	•	•	•		•	**x**	**x**	•	**x**	•
*Myzus* *persicae*	**x**	**x**	•	•		•	•	•	**x**	•
*Sitobion* *avenae*	**x**	•	•	•	•	•	•		•	•
*Pterocomma* *salicis*	•	•	**x**	•	**x**	**x**	**x**	**x**	•	•
*Tuberolachnussalignus*	•	**x**	**x**	**x**	**x**	**x**	**x**	**x**	**x**	**x**
*Metopolophiumdirhodum*	•	•	•	•	•	•	**x**	•	**x**	**x**
*Rhopalosiphumpadi*	**x**	•	**x**	**x**	•	**x**	**x**	**x**	**x**	•
*Nasonoviaribisnigri*	**x**	•	•	**x**	•	**x**	•	•	**x**	**x**
*Aphis fabae*	•	**x**	**x**	**x**	**x**	**x**	**x**	•	**x**	**x**

(X indicates no PCR product, • indicates a PCR product).

### Genomic structure of *A* *. gossypii* OBP and CSP genes

**Figure 3:**
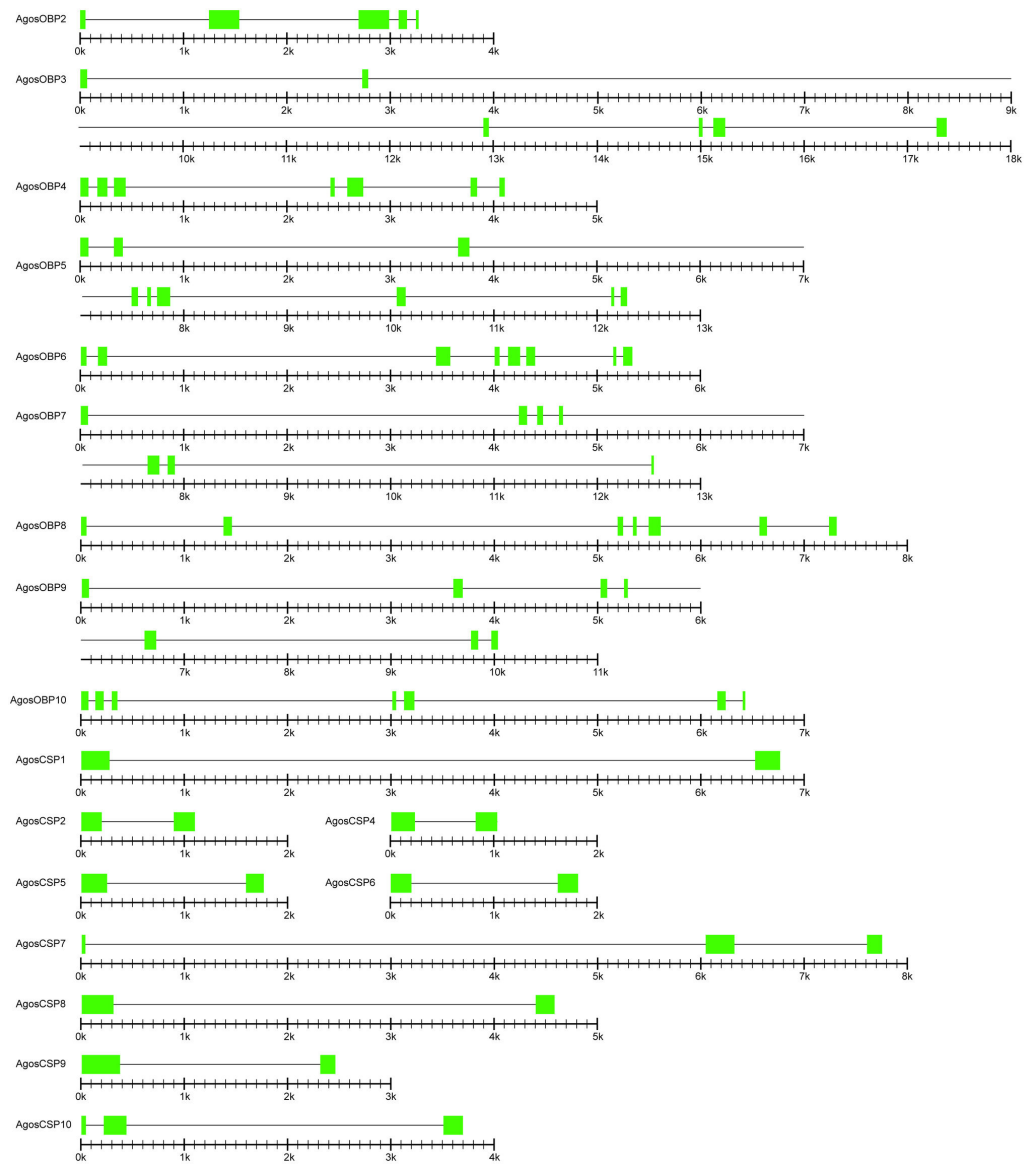
Genomic structure of *A*. ***gossypii* OBP and CSP genes**. The genomic structure of each *A*. *gossypii* *OBP* and *CSP* was analyzed by aligning the mRNA sequence with the genomic DNA sequence using Spidey program. The green rectangles and hairlines represent the extrons and introns, respectively. The scale bars are illustrated under each *A*. *gossypii* *OBP* and *CSP* with every minor mark as 100 bp and every major mark as 1 kb, respectively.

**Figure 4:**
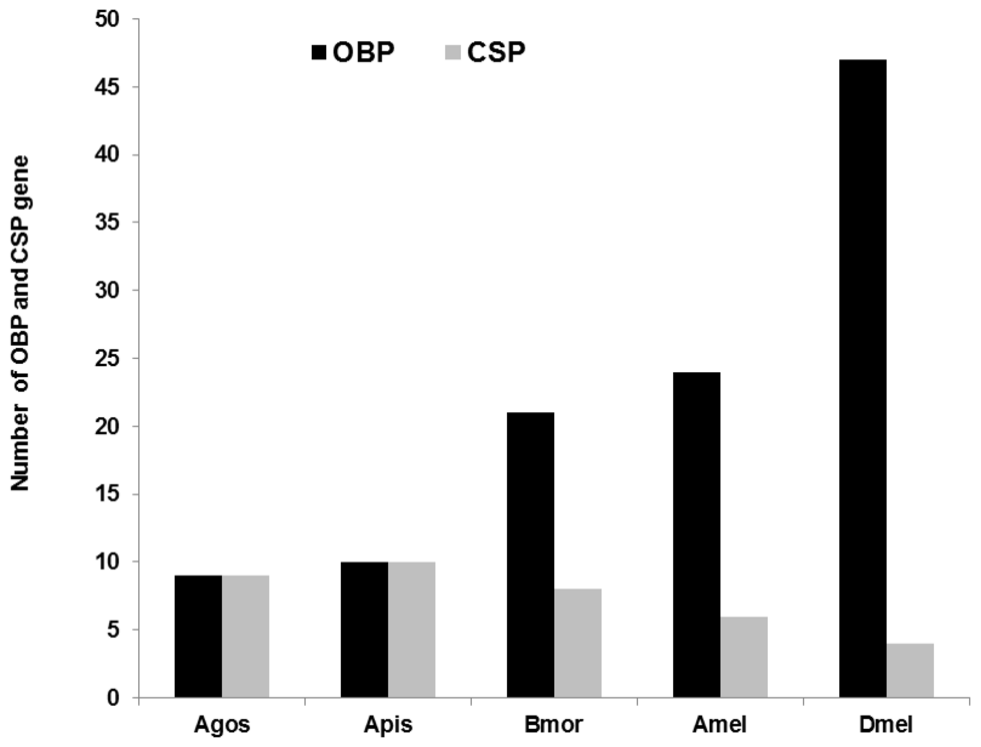
Number of annotated OBP and CSP genes in insect genomes. Agos: *Aphis* *gossypii*. *Apis: Acyrthosiphon pisum*. Bmor: *Bombyx* *mori*. Amel: *Apis* *mellifera*. Dmel: *Drosophila* *melanogaster*.

**Figure 5:**
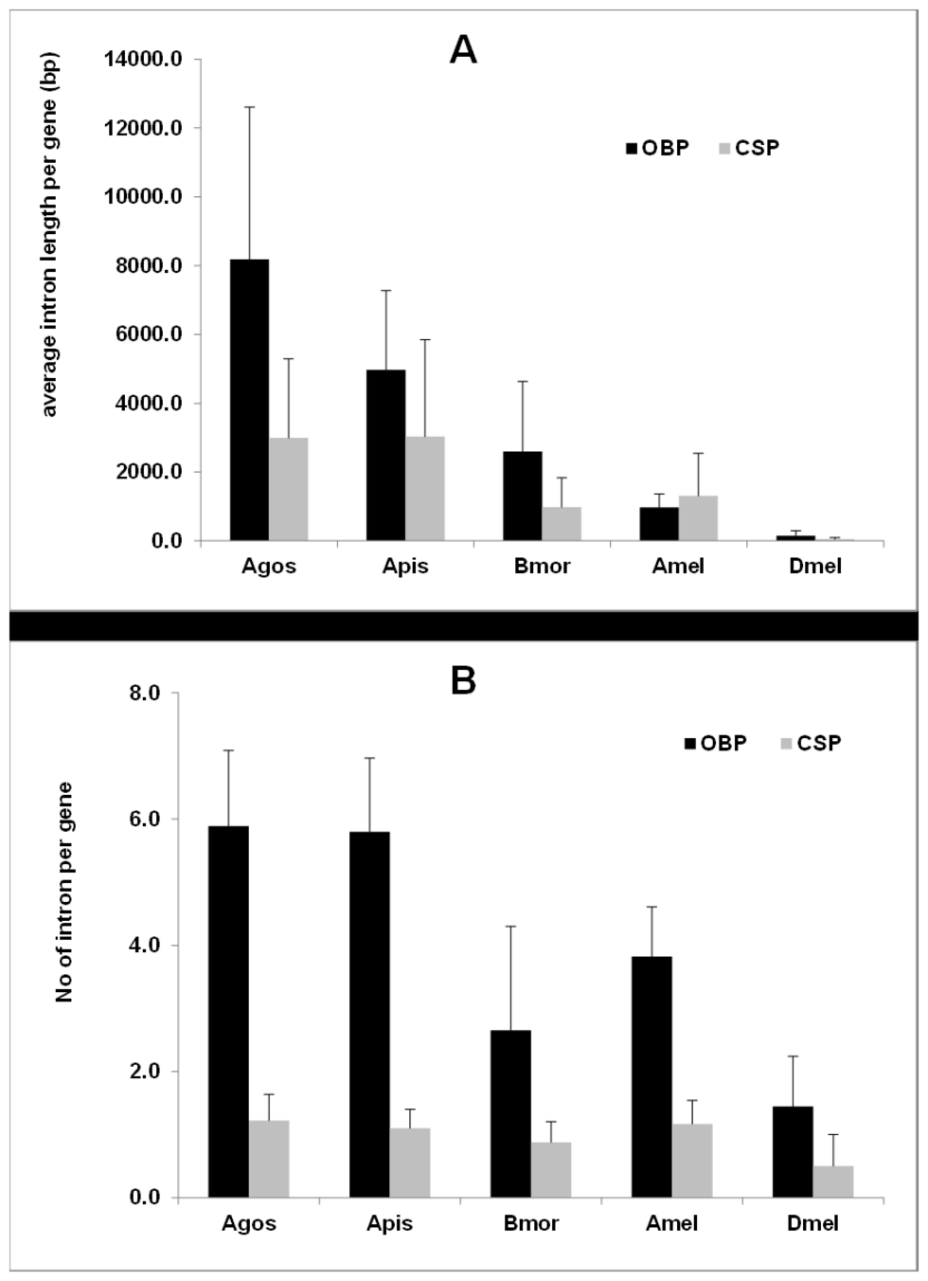
Introns in OBP and CSP genes. (A) intron number, (B) intron length. Agos: *Aphis* *gossypii*. *Apis: Acyrthosiphon pisum*. Bmor: *Bombyx* *mori*. Amel: *Apis* *mellifera*. Dmel: *Drosophila* *melanogaster*.

To validate the putative *A* *. gossypii* OBP and CSP gene annotations and examine their gene structure we cloned them from RNAs by RT-PCR, and sequenced the genomic fragments. The length of the OBP genes ranged from 3.3 kb to 17.4 kb with five (*AgosOBP4, AgosOBP7, AgosOBP8, AgosOBP9* and *AgosOBP10*) having 6 introns and the other four OBP (*AgosOBP2, AgosOBP3, AgosOBP5* and *AgosOBP6*) having 4, 5, 8 and 7 introns, respectively, with an average length ranging from 0.6 kb to 2.0 kb (Figure 3). The CSP genes are much shorter ranging from 1.1 kb to 7.8 kb with either one (*AgosCSP1, AgosCSP2, AgosCSP4, AgosCSP5, AgosCSP6, AgosCSP8* and *AgosCSP9*) or two (*AgosCSP7* and *AgosCSP10*) introns (Figure 3) with the average length of 586 bp to 6250 bp. The number of OBP genes in aphids is much lower than in other insect genomes such as *Drosophila melanogaster* (Figure 4). The intron number and length of the *A* *. gossypii* OBP and CSP genes are consistent with those of the *A. pisum* genes but much higher than those of *D. melanogaster* and other insects (Figure 5). All introns follow the GT-AG rule. Our results suggest that the formation of introns occurred at the early stages of aphid evolution before speciation.

### Expression profiles of *A* *. gossypii* OBP and CSP genes in Morphs and tissue types

**Figure 6:**
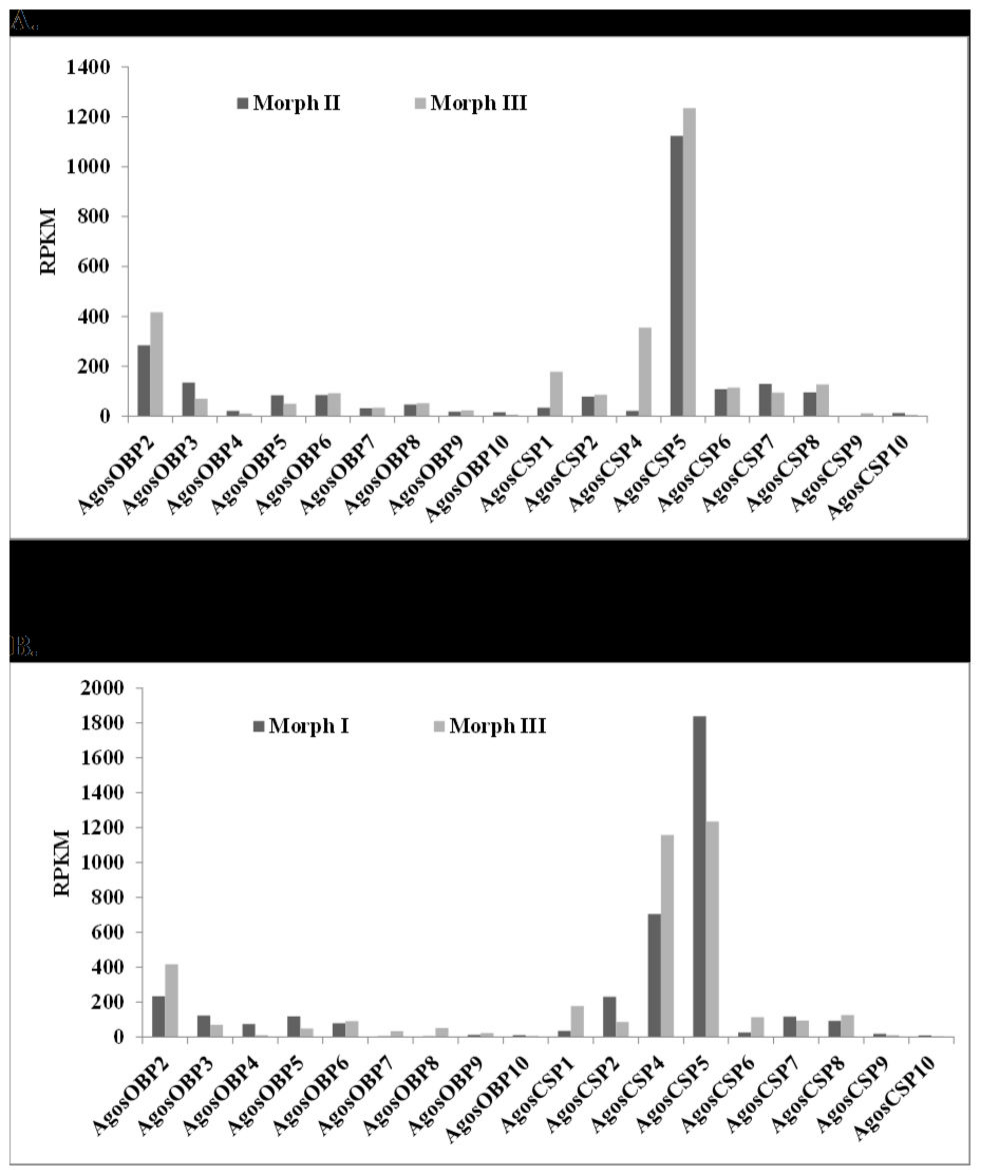
Reads per kilobase pairs per million mapped reads (RPKM). (A) comparison between Morph I and Morph III *A*. *gossypii* (B) comparison between Morph II and Morph III *A*. *gossypii.*

We compared the relative abundance of each *A* *. gossypii* OBP and CSP transcript in the transcriptome dataset between spring wingless form Morphs I and III (Figure 6A) and between summer wingless form Morphs II and winged form Morph III (Figure 6B). Three transcripts, *AgosOBP2*, *AgosCSP1* and *AgosCSP4* are more abundant in Morph III the winged adult form than in either of the wingless forms (Morphs I and II), suggesting a role in the flying phenotype for host search. The OBP2 transcript of the pea aphid *ApisOBP2*, however, is indicated to be expressed at a very high level in the transcriptome libraries of the heads (SRR075802 and SRR075803) and the ovary/embryos (SRR098330) of the pea aphid adult sexuparae, but at very low level in L4 nymphs sexuparae libraries (AphidBase: http://isyip.genouest.org/cgi-bin/gb2/gbrowse/aphidbase/). AgosCSP5 transcripts are the most abundant in all Morphs suggesting a ubiquitous role in *A* *. gossypii* . In the pea aphids CSP4 and CSP5 transcripts are shown to be highly expressed in the male adult library (SRR071347) and the heads (SRR075802 and SRR075803) of the adult sexuparae. In addition ApisCSP4 transcript is also highly expressed in the heads of the parthenogenetic female after 24 hours crowding and solitary treatments (SRR074233 and SRR074231). In contrast, the pea aphid CSP1 is shown to be expressed at very low levels in all 25 transcriptome libraries (AphidBase: http://isyip.genouest.org/cgi-bin/gb2/gbrowse/aphidbase/). Overall the analysis of the cotton aphid transcriptomes shows that apart from OBP2 gene the other OBP genes are expressed at levels lower than the CSPs, in contrast to what is seen in other insects [36,37], suggesting that CSPs may play a more important role in aphids. A wide range of roles have been suggested for CSPs. The first member of the group was reported as being involved in leg regeneration in *Periplaneta* *americana* [38] and a similar protein (olfactory segment-D protein with homology to AgosCSP1) was demonstrated to be specifically expressed in sensilla coeloconica of *D. melanogaster* [39]. Indeed, although many are expressed in the antennae, others are expressed in other tissues including legs [40,41], labial palps [42], tarsi [43], brain [44], proboscis [45], wings [46], the ejaculatory bulb of *D. melanogaster* [39] and the reproductive system of *Locusta* *migratoria* [47] and *Helicoverpa* species [48]. The CSPs expressed in the pheromone gland of the cabbage armyworm, *Mamestra* *brassicae* can bind sex pheromone analogues, suggesting a role in pheromone transport and release [49].

**Figure 7:**
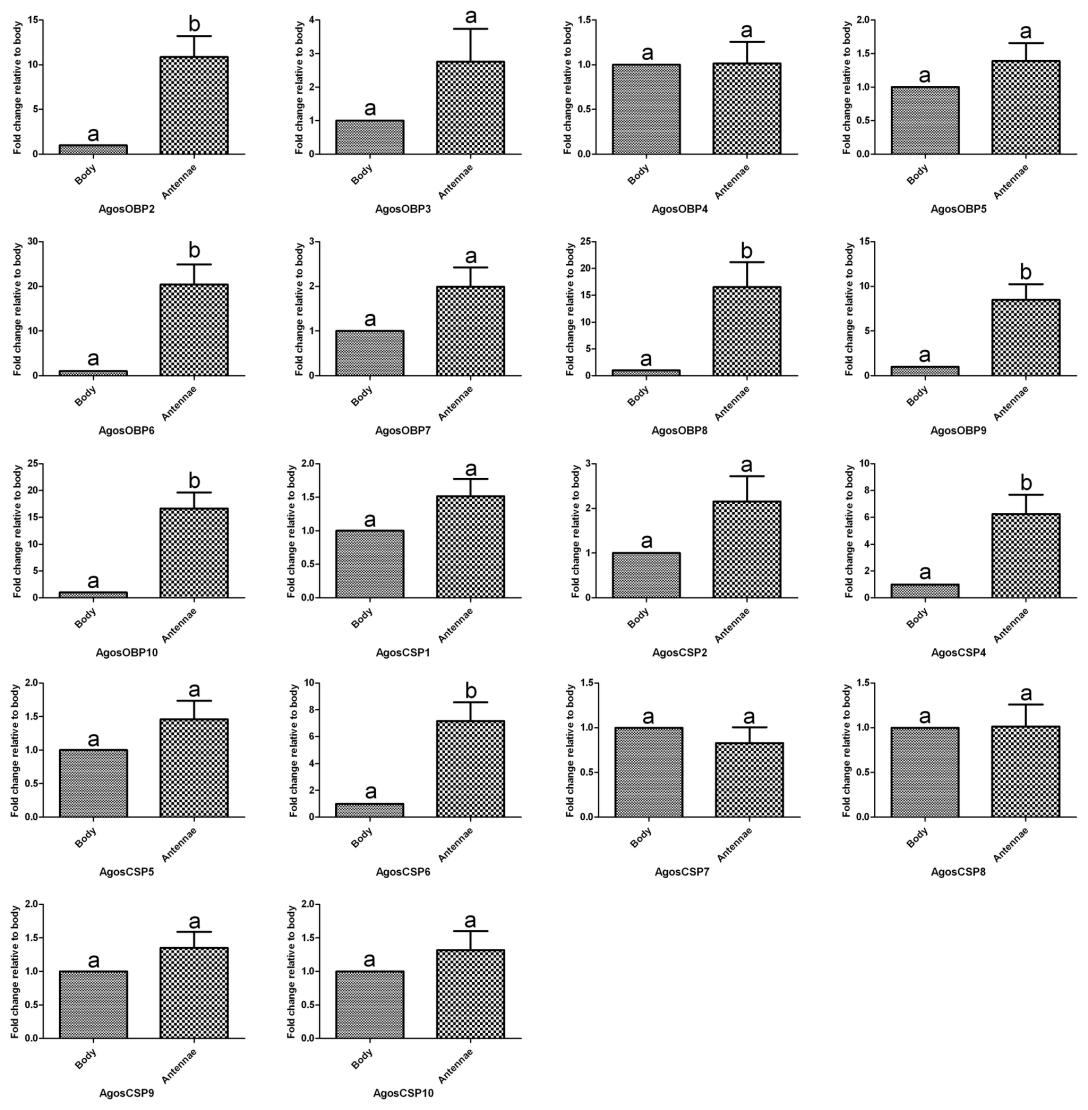
Tissue-specific expression profiles of *A*. ***gossypii* OBPs and CSPs as measured by qRT-PCR**. The fold changes are relative to the transcript levels in the body. Standard errors represented by the error bars, and the letter above each bar indicates the statistical differences; the bars with different letters (a, b, c) indicate significant differences (*p*<0.05) and with same letters indicate no differences between mean expression levels.

**Figure 8:**
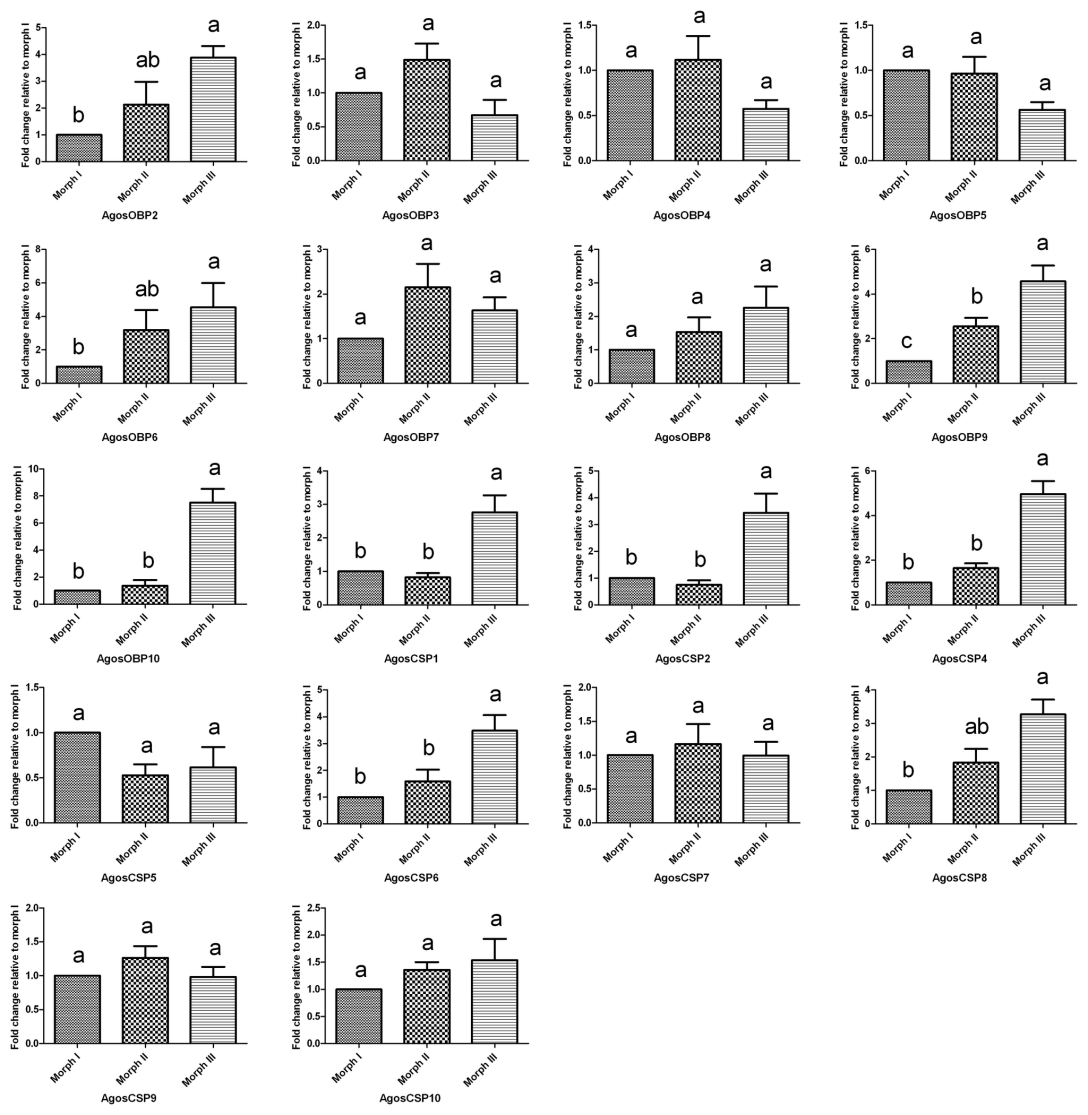
Stage-specific expression profiles of *A*. ***gossypii* OBPs and CSPs as measured by qRT-PCR**. The fold changes are relative to the transcript levels in the Morph I. The standard error is represented by the error bar, and the letter above each bar indicates the statistical differences; the bars with different letters (a, b, c) indicate significant differences (*p*<0.05) and with same letters indicate no differences between mean expression levels.

To further test the role of *A* *. gossypii* OBPs and CSPs in host searching behavior we measured their expression in decapitated bodies and antennae of winged aphids in Morph III by qRT-PCR (Figure 7). This showed that five *A* *. gossypii* *OBPs* (*AgosOBP2, AgosOBP6, AgosOBP8, AgosOBP9* and *AgosOBP10*) and two CSPs (*AgosCSP4, AgosCSP6*) were significantly overexpressed in the antennae compared with the bodies (p<0.05), and five of these (*AgosOBP6, AgosOBP9, AgosOBP10, AgosCSP4* and *AgosCSP6*) were significantly up-regulated in the winged stage (Morph III) compared to both of the wingless Morphs (p<0.05) (Figure 8). Up regulation in antennae and the winged stage may indicate their participation in cotton aphid olfaction during attraction to the winter hosts and may offer targets for disrupting this activity.

In addition three other CSP genes (*AgosCSP1, AgosCSP2* and *AgosCSP8*) were significantly up-regulated in the winged aphids although expressed at a similar level in bodies and antennae. These may play a role other than in olfaction in the physiology and ecology of *A* *. gossypii* perhaps as carriers to capture, release, transport and protect hydrophobic molecules, for example, during sex pheromone production.

## Conclusions

This study has identified OBPs and CSPs in the cotton aphid *A* *. gossypii* and shown that these proteins are clustered in highly conserved groups comprising OBP genes from different aphid species. The genes have more and longer introns than in non-aphid species suggesting different evolution mechanisms from those of other insects. The overexpression of some OBP and CSP genes in the antennae and winged adults produced when the cotton aphids are ready to migrate in search of new hosts suggests that they play a role in host location and may offer a target for intervention to prevent completion of the life-cycle. This study provides, for the first time, the antennal expression profile of aphid OBP and CSP transcripts and three morph stages with ecological significance from a population composed of genetically identical individuals derived parthenogenetically from a single founding aphid. Some transcripts (*AgosOBP2, AgosOBP8, AgosCSP4,* and *AgosCSP6*) that are highly expressed in the cotton aphid antennae have homologues that are indicated to be expressed highly in the male library and the sexuparae head libraries of the pea aphid. Further studies are needed to see which olfactory cues (plant volatiles and/or sex pheromones) may be perceived by these proteins. However, the homologues of all up-regulated transcripts in the winged morphs of the cotton aphid (*AgosOBP6, AgosOBP9, AgosOBP10, AgosCSP1, AgosCSP2, AgosCSP4, AgosCSP6* and *AgosCSP8*) have very low abundance in the winged female transcript library (SRR073136) of the pea aphid reported in AphidBase. Since the experimental size is relative small (3 technical replicates and 2 biological replicates) and all individuals are expected to be genetically homogeneous, the expression profiling between tissue types may be regarded from "one individual" and the results might not be representative of the entire species/population. Further statistical analyses on the pea aphid RNA-seq data in AphidBase are needed to confirm such comparative results. Nevertheless this expression difference between the cotton and pea aphids and the differential expression among different aphid species of 15 OBP and 13 CSP transcripts annotated in the pea aphid genome demonstrate a significant regulation of these olfactory genes in aphid species, thus indicating the important role they may play in aphid physiology.

## Supporting Information

Table S1 **Gene specific primers used for the cloning of the open reading frames of *A* *. gossypii* *OBP* and *CSP* genes.**
(DOCX)

Table S2 **Primers used in real-time PCR for determination of expression levels of *A* *. gossypii* *OBP* and *CSP* genes.**
(DOCX)

Table S3 **A percent identity matrix of *A* *. gossypii* OBPs.**
(DOCX)

Table S4 **A percent identity matrix of *A* *. gossypii* CSPs.**
(DOCX)

Figure S1 **Alignment of the *A* *. gossypii* OBPs.** Full-length amino acid sequences of AgosOBP2-10 were aligned by ClustalX 2,1. Green boxes show conserved cysteine residues. Accession numbers are listed in Table 1.
(DOCX)

Figure S2 **Alignment of the *A* *. gossypii* CSPs.** Full-length amino acid sequences of AgosCSPs were aligned by ClustalX 2.1. Green boxes show conserved cysteine residues. Accession numbers are listed in Table 1.
(DOCX)

Figure S3 **Phylogenetic tree of CSPs from *A* *. gossypii* and *A* *. pisum* .** Numbers on branches show values of 1000 times replication bootstrap analysis and the bootstrap values are listed at each node. Agos, *Aphis* *gossypii*; Apis, *Acyrthosiphon pisum*. The accession numbers of AgosCSPs are listed in Table 3, the accession numbers of ApisCSP1-10 are NP_001119650, NP_001119651, NP_001128404, NP_001119652, NP_001119649, NP_001156287, NP_001156200, XP_001951447, XP_001948415 and XP_001947629, respectively.
(DOCX)
