# Supplementary material for: Identification and Expression Profiling of Odorant Binding Proteins and Chemosensory Proteins between Two Wingless Morphs and a Winged Morph of the Cotton Aphid Aphis gossypii Glover
Source: PLoS One. 2013 Sep 20;8(9):e73524. doi: 10.1371/journal.pone.0073524 (PMC3779235; doi:10.1371/journal.pone.0073524)
Supplement: Table S1 — Gene specific primers used for the cloning of the open reading frames of A . gossypii OBP and CSP genes. (DOCX) [file pone.0073524.s001.docx]

**Supplementary Table S1**. Gene specific primers used for the cloning of the open reading frames of *A. gossypii* *OBP* and *CSP* genes.

| Primer name | Sequence (5'-3') | Tm (℃) |
| --- | --- | --- |
| OBP2-Forward | ATGAAGGTATCTGCAGCGACC | 59.3 |
| OBP2-Reverse | TTATGCTTTAGGGAAGAAATTGATTT | 60.1 |
| OBP3-Forward | ATGATTTCGTCAACTTTCTACACATC | 59.1 |
| OBP3-Reverse | TCAAGTTGAATTGTCAAGTTTCAACT | 59.7 |
| OBP4-Forward | ATGCGTGGAAATTATTCTTTGGT | 60.0 |
| OBP4-Reverse | TTAAACTTGACGTTGGCTTAAGTTATT | 60.4 |
| OBP5-Forward | ATGAAAATGTCCGCTAACGGTG | 61.8 |
| OBP5-Reverse | TCATTGGTTTGATGGTGATTTTTG | 61.7 |
| OBP6-Forward | ATGCAAAAAGTGGTTTTTCTATGTATT | 60.1 |
| OBP6-Reverse | TTAAATTAATTTAGGTGGTGATTGGAA | 60.7 |
| OBP7-Forward | ATGAATATGTTACCAGCCACTGTTTT | 60.9 |
| OBP7-Reverse | CTAGAGTGGTAGAAATTCTAAACTTTTGG | 60.4 |
| OBP8-Forward | ATGTTCGCTTTTAAAGTGGCG | 60.1 |
| OBP8-Reverse | TTATACGATGCTGTGTCTGAATTTCG | 62.9 |
| OBP9-Forward | ATGATAATCAAAAAGACGTTGTTGG | 59.9 |
| OBP9-Reverse | TTATTTCGATTTTGGTTTCATCTTC | 59.4 |
| OBP10-Forward | ATGGAACATTTACGTGGTACAAACG | 62.5 |
| OBP10-Reverse | TCATTGTAATGGTAGTAGTTCGATAGTCA | 60.7 |
| CSP1-Forward | ATGAATATTTTAACGATTTTTTGTTATGT | 59.2 |
| CSP1-Reverse | CTAAATATTCCGCTTTTTTTTAGTAGTATC | 59.8 |
| CSP2-Forward | ATGGCGCATCTTAACTTATTTGTC | 60.1 |
| CSP2-Reverse | TTAAGCTTTAAGAGGTTTAGCTGAAGC | 61.7 |
| CSP4-Forward | ATGGATTCCAGAATTGCAGTAGTCT | 60.7 |
| CSP4-Reverse | TTAAAATTTAGTAAAACCCTTTTTCTTTTC | 61.1 |
| CSP5-Forward | ATGCACTGCAAGGTTTTGATCG | 62.6 |
| CSP5-Reverse | TTACGCGTCGAGGAACTTGTT | 60.1 |
| CSP6-Forward | ATGATCAAACTCATTCTAGCAATTGC | 61.4 |
| CSP6-Reverse | TTAAACATTAATGTTCAATTTTTTTGCT | 60.5 |
| CSP7-Forward | ATGTCTCGATCGTCGTCAAGTG | 59.9 |
| CSP7-Reverse | TTAAAATCCGCTCTGGTACTGTTTC | 61.5 |
| CSP8-Forward | ATGAACAATATTATAATGAACAATTCGC | 60.4 |
| CSP8-Reverse | CTAATGGTTCATGAAAGCTAGAATCTC | 60.0 |
| CSP9-Forward | ATGTCAGCGTTTTGCCTGAACT | 61.5 |
| CSP9-Reverse | TCATATTTTTTTTTTATAAATTGGTTCGA | 61.7 |
| CSP10-Forward | ATGATCAACACTCGACCTCGGA | 61.7 |
| CSP10-Reverse | TTATTTCGATTCGTTTAAGTTTTCTAAG | 59.8 |
|  |  |  |
