# Supplementary material for: Identification and Expression Profiling of Odorant Binding Proteins and Chemosensory Proteins between Two Wingless Morphs and a Winged Morph of the Cotton Aphid Aphis gossypii Glover
Source: PLoS One. 2013 Sep 20;8(9):e73524. doi: 10.1371/journal.pone.0073524 (PMC3779235; doi:10.1371/journal.pone.0073524)
Supplement: Table S2 — Primers used in real-time PCR for determination of expression levels of A . gossypii OBP and CSP genes. (DOCX) [file pone.0073524.s002.docx]

**Supplementary Table S2**. Primers used in real-time PCR for determination of expression levels of *A. gossypii* *OBP* and *CSP* genes.

| Primer name | Sequence (5'-3') | Position (bp) |
| --- | --- | --- |
| OBP2-Forward | CAACACCAAAGCCAACAATG | 323-342 |
| OBP2-Reverse | GACCAGTGCCATGATTTCCT | 419-438 |
| OBP3-Forward | GCGGGGTTGAAGAAATACTG | 253-272 |
| OBP3-Reverse | TTCAAGCACGCCATCACTAC | 376-395 |
| OBP4-Forward | GCTTTCCGGTAGTTGATGGT | 338-357 |
| OBP4-Reverse | TTGCTGTGCGATGGAAATAC | 431-450 |
| OBP5-Forward | CTAACGGTGCAACGATGAAA | 14-33 |
| OBP5-Reverse | GTCGCTGTCTTCTGTGTCCA | 116-135 |
| OBP6-Forward | TTGGACAATGCTTGGGAAGT | 187-206 |
| OBP6-Reverse | TGGTGGCTCATTTTCGTGTA | 269-288 |
| OBP7-Forward | TCCCGAGAACAACAACAACA | 180-199 |
| OBP7-Reverse | GCCAACATCGTCATCTTGTCT | 279-299 |
| OBP8-Forward | TTTCGGTTGAAGAGGCAACT | 239-258 |
| OBP8-Reverse | CCAACGCACACTCTTCAGTC | 351-370 |
| OBP9-Forward | TTTTCGGTTGCATGTTTTCA | 41-60 |
| OBP9-Reverse | CCCCAATCAGCATCCTTAAA | 139-158 |
| OBP10-Forward | GCAGCAATGGCAGTAGACAA | 277-296 |
| OBP10-Reverse | TGCAGCTTCACAACCATCTT | 359-378 |
| CSP1-Forward | GTGCGACACTCAAGTCAAGC | 39-58 |
| CSP1-Reverse | TGACGACGTTTCTCTGATGG | 128-147 |
| CSP2-Forward | TTCAGCAGCGGAAGAAAAGT | 54-73 |
| CSP2-Reverse | TTTCCTTCGTCAAGCAAACA | 148-167 |
| CSP4-Forward | GCCACAAAAAGATGCCGTAG | 69-88 |
| CSP4-Reverse | TTGTCCAACAGACATTGAACG | 174-194 |
| CSP5-Forward | GGTTTTGATCGCTCTTTGCT | 12-31 |
| CSP5-Reverse | TGTTCGACGTTGAGCTTGTC | 139-158 |
| CSP6-Forward | GACAGCCCCGGCTAAATATAC | 60-80 |
| CSP6-Reverse | TTTTCCCGTTTCCATCAGAC | 152-171 |
| CSP7-Forward | ATCGTCGTCAAGTGTCACCA | 9-28 |
| CSP7-Reverse | TGCCGCTAAGGTTTCAGATT | 116-135 |
| CSP8-Forward | CGCTGTTAGCCGTTACGATT | 50-69 |
| CSP8-Reverse | AACATCATCGTCTGCTGCTG | 145-164 |
| CSP9-Forward | GTCAGCGTTTTGCCTGAACT | 3-22 |
| CSP9-Reverse | TCGTCGGTATCCCTTTTGAC | 121-140 |
| CSP10-Forward | ACACTCGACCTCGGAAATTG | 8-27 |
| CSP10-Reverse | TTCTCGGTTGACCAGATGAG | 107-126 |
| β-actin-Forward | GGGAGTCATGGTTGGTATGG | 126-145 |
| β-actin-Reverse | TCCATATCGTCCCAGTTGGT | 232-251 |
| 18SRNA-Forward | AGTGAGGTCTTCGGACTGGA | 2332-2351 |
| 18SRNA-Reverse | CCTACGGAAGCCTTGTTACG | 2437-2456 |
