# Supplementary material for: Identification and Expression Profiling of Odorant Binding Proteins and Chemosensory Proteins between Two Wingless Morphs and a Winged Morph of the Cotton Aphid Aphis gossypii Glover
Source: PLoS One. 2013 Sep 20;8(9):e73524. doi: 10.1371/journal.pone.0073524 (PMC3779235; doi:10.1371/journal.pone.0073524)
Supplement: Table S3 — A percent identity matrix of A . gossypii OBPs. (DOCX) [file pone.0073524.s003.docx]

**Supplementary Table S3. A percent identity matrix of** ***A. gossypii* OBPs**

|  | AgosOBP2 | AgosOBP3 | AgosOBP4 | AgosOBP5 | AgosOBP6 | AgosOBP7 | AgosOBP8 | AgosOBP9 | AgosOBP10 |
| --- | --- | --- | --- | --- | --- | --- | --- | --- | --- |
| AgosOBP2 | 100 |  |  |  |  |  |  |  |  |
| AgosOBP3 | 12 | 100 |  |  |  |  |  |  |  |
| AgosOBP4 | 12 | 13 | 100 |  |  |  |  |  |  |
| AgosOBP5 | 15 | 13 | 11 | 100 |  |  |  |  |  |
| AgosOBP6 | 9 | 15 | 12 | 14 | 100 |  |  |  |  |
| AgosOBP7 | 17 | 11 | 9 | 9 | 12 | 100 |  |  |  |
| AgosOBP8 | 22 | 15 | 13 | 10 | 10 | 11 | 100 |  |  |
| AgosOBP9 | 10 | 18 | 14 | 10 | 13 | 16 | 13 | 100 |  |
| AgosOBP10 | 16 | 15 | 16 | 13 | 14 | 24 | 14 | 14 | 100 |

The calculations are based on the alignment of amino acid sequence by Vector NTI. The percentage of identity of each pair is shown.
