# Supplementary material for: Identification and Expression Profiling of Odorant Binding Proteins and Chemosensory Proteins between Two Wingless Morphs and a Winged Morph of the Cotton Aphid Aphis gossypii Glover
Source: PLoS One. 2013 Sep 20;8(9):e73524. doi: 10.1371/journal.pone.0073524 (PMC3779235; doi:10.1371/journal.pone.0073524)
Supplement: Table S4 — A percent identity matrix of A . gossypii CSPs. (DOCX) [file pone.0073524.s004.docx]

**Supplementary Table S4. A percent identity matrix of *A. gossypii* CSPs.**

|  | AgosCSP1 | AgosCSP2 | AgosCSP3 | AgosCSP4 | AgosCSP5 | AgosCSP6 | AgosCSP7 | AgosCSP8 | AgosCSP9 |
| --- | --- | --- | --- | --- | --- | --- | --- | --- | --- |
| AgosCSP1 | 100 |  |  |  |  |  |  |  |  |
| AgosCSP2 | 28 | 100 |  |  |  |  |  |  |  |
| AgosCSP3 | 20 | 27 | 100 |  |  |  |  |  |  |
| AgosCSP4 | 28 | 37 | 22 | 100 |  |  |  |  |  |
| AgosCSP5 | 27 | 32 | 28 | 26 | 100 |  |  |  |  |
| AgosCSP6 | 28 | 38 | 23 | 30 | 28 | 100 |  |  |  |
| AgosCSP7 | 15 | 22 | 18 | 18 | 19 | 16 | 100 |  |  |
| AgosCSP8 | 21 | 31 | 27 | 23 | 25 | 26 | 19 | 100 |  |
| AgosCSP9 | 19 | 18 | 12 | 17 | 21 | 18 | 22 | 18 | 100 |

The calculations are based on the alignment of amino acid sequence by Vector NTI. The percentage of identity of each pair is shown.
