# Supplementary material for: Identification and Expression Profiling of Odorant Binding Proteins and Chemosensory Proteins between Two Wingless Morphs and a Winged Morph of the Cotton Aphid Aphis gossypii Glover
Source: PLoS One. 2013 Sep 20;8(9):e73524. doi: 10.1371/journal.pone.0073524 (PMC3779235; doi:10.1371/journal.pone.0073524)

**Figure S2. Alignment of the *A. gossypii* CSPs**. Full-length amino acid sequences of AgosCSPs were aligned by ClustalX 2.1. Green boxes show conserved cysteine residues. Accession numbers are listed in Table 3.


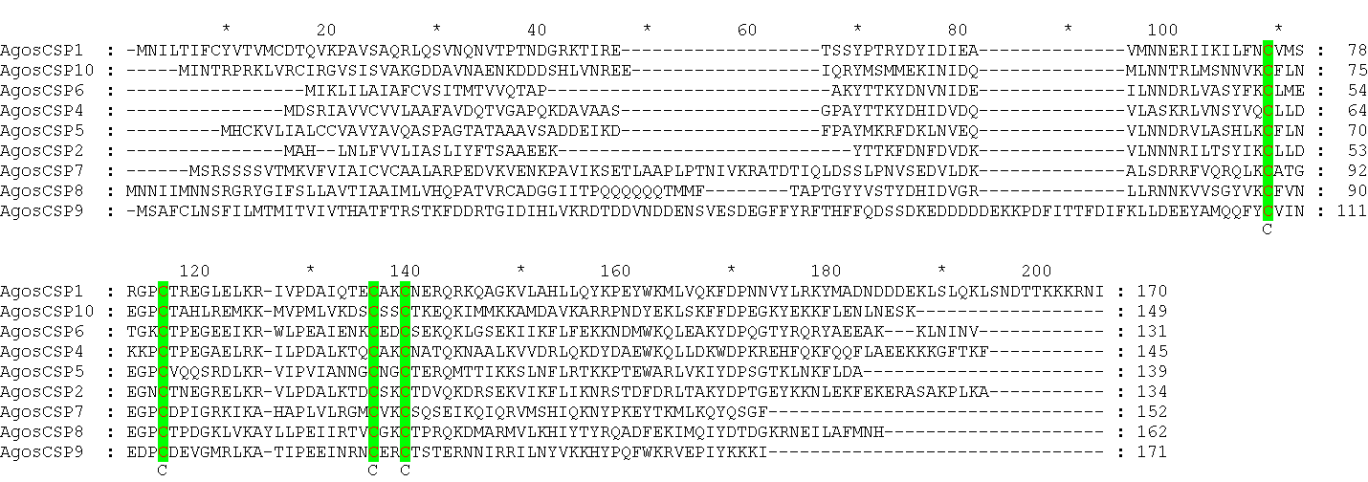

Supplement: Figure S2 — Alignment of the A . gossypii CSPs. Full-length amino acid sequences of AgosCSPs were aligned by ClustalX 2.1. Green boxes show conserved cysteine residues. Accession numbers are listed in Table 1. (DOCX) [file pone.0073524.s006.docx]
