# Supplementary material for: Identification and Expression Profiling of Odorant Binding Proteins and Chemosensory Proteins between Two Wingless Morphs and a Winged Morph of the Cotton Aphid Aphis gossypii Glover
Source: PLoS One. 2013 Sep 20;8(9):e73524. doi: 10.1371/journal.pone.0073524 (PMC3779235; doi:10.1371/journal.pone.0073524)
Supplement: Figure S3 — Phylogenetic tree of CSPs from A . gossypii and A . pisum . Numbers on branches show values of 1000 times replication bootstrap analysis and the bootstrap values are listed at each node. Agos, Aphis gossypii; Apis, Acyrthosiphon pisum. The accession numbers of AgosCSPs are listed in Table 3, the accession numbers of ApisCSP1-10 are NP_001119650, NP_001119651, NP_001128404, NP_001119652, NP_001119649, NP_001156287, NP_001156200, XP_001951447, XP_001948415 and XP_001947629, respectively. (DOCX) [file pone.0073524.s007.docx]

**Figure S3. Phylogenetic tree of CSPs from *A. gossypii* and *A. pisum*** . Numbers on branches show values of 1000 times replication bootstrap analysis and the bootstrap values are listed at each node. Agos, *Aphis gossypii*; Apis, *Acyrthosiphon pisum*. The accession numbers of AgosCSPs are listed in Table 3, the accession numbers of ApisCSP1-10 are NP_001119650, NP_001119651, NP_001128404, NP_001119652, NP_001119649, NP_001156287, NP_001156200, XP_001951447, XP_001948415 and XP_001947629, respectively.
